# Contagious Pleuro-pneumonia

**Published:** 1895-05

**Authors:** 


Groundless Reports of Contagious Pleuro-pneumonia.—The unfortunate report that reached the public press and was cabled abroad of the suspected outbreak of "contagious pleuro-pneumonia" in Kansas should be a very forcible lesson to the veterinarians of our country. This unfortunate error is one that might be repeated at any time, and should impress very forcibly upon the minds of the veterinarians the great responsibility and trust that rests upon their shoulders, and is conveyed by invested gigantic monetary interests to the whole country, and should be a strong reminder that in all their inspections the greatest care and caution should always be exercised. While this applies in general, it specially applies in regard to those contagious and infectious diseases that have found lodgment on our shores from time to time by importation from other countries. At this time it was a peculiar misfortune that the slightest suspicion of the existence of such a disease should be announced when other countries had placed embargoes against our cattle on the grounds of the existence of "contagious pleuro-pneumonia" among our live stock, and the most thorough inspection should always precede judgment upon such questions, in order that no mistakes may be made.

We publish in connection with this reported outbreak of "contagious pleuro-pneumonia" a statement from Dr. N. S. Mayo, whose name was used in connection with the announcement of the existence of this disease through Associated Press news; and, likewise, a report of those who participated in the subsequent investigation that demonstrated beyond any question the non-existence of this disease. In fact, it can be said with absolute surety that this disease has never existed west of the Mississippi river, and has not for nearly three years been known to exist, either in the chronic or acute form, in any section of this country.

Under date of March 19 the report that South Dakota had quarantined against Texas on the grounds of "contagious pleuro-pneumonia" seemed to have been based on absolutely wrong deductions by the Governor of that State. Contagious pleuro-pneumonia has never existed in that section of our country at any time, there never having been a single recorded case in that territory. Such movements as this are only destined to embarrass the National Government at Washington in endeavoring to have lifted the embargoes made against us in foreign countries on the grounds of the existence of this disease in our territory. It is only necessary to repeat the fact that not a single case of chronic or acute "contagious pleuro-pneumonia" has been known in the United States for the past three years, and the most exhaustive examination has been made to determine this point.

We publish in another portion of the JOURNAL a statement of the reported outbreak in Kansas, which also proves to be another grievous error.

